# Exploring physiotherapists’ personality traits that may influence treatment outcome in patients with chronic diseases: a cohort study

**DOI:** 10.1186/s12913-015-1225-1

**Published:** 2015-12-16

**Authors:** Elisah Margretha Buining, Margit K. Kooijman, Ilse C. S. Swinkels, Martijn F. Pisters, Cindy Veenhof

**Affiliations:** Physiotherapy Science, Program in Clinical Health Sciences & Department of Rehabilitation, Nursing Science and Sport, Brain Center Rudolf Magnus, University Medical Center Utrecht, Utrecht, The Netherlands; NIVEL, Netherlands Institute for Health Services Research, PO Box 1568, Utrecht, 3500 BN The Netherlands; Center for Physical Therapy Research and Innovation in Primary Care, Julius Health Care Centers, Utrecht, The Netherlands; Department of Rehabilitation, Nursing Science & Sport, University Medical Center Utrecht, Brain Center Rudolf Magnus, Utrecht, 3508 GA The Netherlands

**Keywords:** Therapist effects, Chronic diseases, Personality, Neuroticism, Big five, Physiotherapy, Osteoarthritis

## Abstract

**Background:**

During treatment of patients with Chronic Diseases (CD) the therapist-patient interaction is often intense, and the strategies used during treatment require physiotherapists to assume a coaching role. Uncovering therapist factors that explain inter-therapist variation might provide tools to improve treatment outcome and to train future therapists. The purpose of this study was to explore the so-called ‘therapist-effect’, by looking at the influence of intrinsic therapist factors, specifically personality traits, on treatment outcome in patients with CD.

**Methods:**

A cohort study was performed using data from the NIVEL Primary Care Database (NPCD) in 2011–2012 and an additional questionnaire. Patients with CD (*n* = 393) treated by Dutch physiotherapists working in outpatient practices (*n* = 39) were included. Patient and treatment outcome variables were extracted from NPCD. The course of complaint was measured using the Numeric Rating Scale. Therapist variables were measured using a questionnaire consisting of demographics and the Big Five traits: Extraversion, Neuroticism, Agreeableness, Conscientiousness and Openness to experiences. Data were analysed using multilevel linear regression.

**Results:**

Only Neuroticism was found to be significant (Neuroticism F = 0.71, *P* = 0.01; therapist gender F = 0.72, *P* = 0.03; life events F = −0.54, *P* = 0.09; patient gender F = −0.43, *P* = 0.10; patient age F = 0.01, *P* = 0.27). Subgroup analyses of 180 patients with Osteoarthritis and 30 therapists showed similar results.

**Conclusions:**

There are indications that patients with CD who are treated by therapists who tend to be calmer, more relaxed, secure and resilient have a greater reduction in severity of complaints compared to patients treated by therapists who show less of these traits. Being a male therapist and having experienced life events influence outcome positively. However, more extensive research is needed to validate the current findings.

## Background

Chronic diseases (CDs) are a growing health problem worldwide, causing 89 % of all mortality in the Dutch population in 2014 [1]. As CDs, such as cardiovascular diseases, cancers, chronic respiratory diseases, arthritis and diabetes, are generally of long duration and low progression, patients need ongoing management over a period of months, years or decades. Besides this, patients with CD generally need more healthcare than patients with non-CD [2]. In daily physiotherapy practice, treatment sessions are often prolonged compared to patients with non-CD [3]. Considerable research has gone into how to treat patients with CD in daily physiotherapy practice. This information forms the basis of Dutch physiotherapy evidence-based statements and guidelines regarding these diseases [4–9]. In these guidelines the core components of treatment are similar: (1) patients learn to manage and live with their disease in daily life and (2) they learn how to become and stay physically fit [10]. Both cases require a change in the patients’ behaviour and a need to adopt the skill of self-management.

Research by Lewis and colleagues [11] shows that physiotherapists can influence treatment outcome. In their study comparing two randomized clinical trials (RCTs) therapists accounted for around 3–7 % of the overall effect in patient disability outcome scores. The use of strategies to direct behavioural change and self-management within treatment requires physiotherapist to adopt a coaching role [4–10]. In addition, the prolonged therapy sessions lead to more contact with the treating party. Lewis et al. [11] hypothesized that an approach focusing on coaching may contribute to the effect of therapists on treatment outcomes. Based on these considerations, we assume that therapist-patient interaction is more intense in the treatment of patients with CD and therefore treatment outcome might be subject to greater influence by therapist related factors: the so-called ‘therapist effect’.

Identifying therapist related factors that affect treatment outcome could provide tools to improve treatment outcome in patients with CD. Some research has gone into extrinsic therapist related factors such as physiotherapists’ experience and education, [11–18] showing no consistent influence on patient outcome. Only organizational related stress was associated with better physical patient outcomes. Unfortunately, the study’s conclusions are limited due to it being a cross-sectional analysis - time and influences at different hierarchical level were not taken into account [19]. Although proposed, [12, 15, 18] rather less attention has been paid to exploring intrinsic therapist factors such as personal beliefs, calmness or empathy.

The influence of intrinsic healthcare professionals’ characteristics on treatment outcome has been studied in related professional fields. Boerebach et al. [20] conducted a systematic review in which they examined the influence of clinicians’ personality and interpersonal behaviour on the quality of patient care. However, based on the low number of studies found, they could give no conclusion regarding the effect of personality on patient care. In their study, four articles were found showing a small effect of ‘Openness to experience’ [21], no effect of ‘Agreeableness’ , Openness to experiences’ [22, 23] or ‘Extraversion’ [24], and inconsistent findings for ‘Neuroticism’ and ‘Conscientiousness’ [22–24]. In a sample of patients with anxiety and mood disorders, Heinonen et al. [25] showed that active, engaging and extrovert psychotherapists achieved a faster symptom reduction in short-term treatment while more cautious, non-intrusive therapists realized greater benefits during long-term treatment. Also, treatments by psychotherapists who had lower confidence and did not enjoy their work predicted poorer outcomes on the short- and long-term [25]. In four studies, [26–29] more empathic psychotherapists and general practitioners affected treatment outcome in a positive manner.

A systematic approach to examining intrinsic physiotherapist factors is to look at personality traits, as contained in the Big Five personality theory [30, 31]. The Big Five is a widely used and accepted approach to examining the structure of inter-individual differences, using five personality dimensions. Based on prior theoretical research such as psycholexical theory [32], these personality dimensions have been shown to closely reflect actual behaviour traits [33]. Greater understanding of the influence of personality traits may contribute to general understanding of the physiotherapist effect and might be useful for general training of therapists. To our knowledge, no study has investigated the influence of physiotherapists’ personality traits on treatment outcome in patients with CD. Therefore, the objective of this study is to explore the influence of physiotherapists’ personality traits, using the Big Five, on treatment outcome in patients with CD in primary care.

## Methods

### Design overview

For this study, data were used from the NIVEL Primary Care Database (NPCD). This longitudinal registration database holds data of several primary care health care providers, including physiotherapists. NPCD contains information on the domains patients’ demographics, treatment plan, treatment and evaluation [34]. Data are continuously collected in a representative network of 73 therapists working in 40 primary care physical therapy practices. The therapists included worked at least 50 % of their hours as a general physiotherapist in primary care practices. Patients were recruited using a convenience sample. All patients treated by therapists who participated in NPCD were eligible to participate and were registered in the database, with the exception of those who declined to participate. However, this rarely occurred. Data were extracted monthly from the electronic medical records used to reimburse treatment costs. In addition, the therapists completed an online questionnaire annually. Informed consent was not applicable, as the study does not fall within the scope of the Medical Research Involving Subjects Act. However, the study did adhere to the Declaration of Helsinki [35]. Specifics regarding the method are reported by Swinkels et al. [36–38].

### Study setting and design

Data related to physiotherapists who participated in the NPCD period 2009–2011 were obtained by entering additional questions on the annual NPCD-physical therapy questionnaire. The additional questions concerned therapists’ experience of a life-event and their personality traits, using the Big Five Inventory (BFI) [39–41]. The questionnaire was sent digitally to 73 therapists in February 2012. To reduce non-response, two reminders were sent digitally to non-responding therapists 10 and 20 days after the questionnaire was provided.

This study used patient data from the NPCD period 2009–2011. The registration period of three years was chosen for practical reasons related to sample size and treatment duration of CD patients. Physiotherapists collected patients’ demographics at the start of treatment. Information regarding the course of complaints was collected at the start and end of therapy.

### Sample

**Fig. 1:**
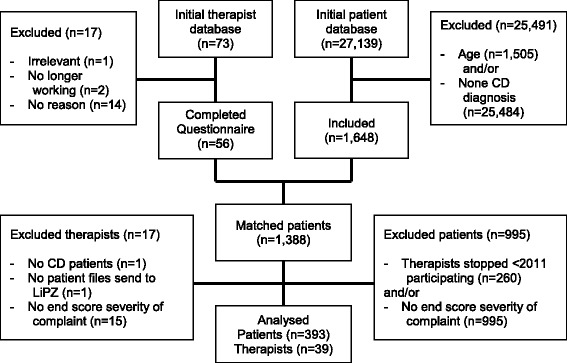
Flow diagram of therapist and patient selection

All therapists who participated in NPCD were included, with the exception of those who had stopped participating by 2011. NPCD registered patients were eligible if they were adults (≥18 years) who started treatment in the period 2009–2011, with CDs defined as non-reversible, non-communicable, diseases [42]. The patient’s diagnosis was registered by the physiotherapist according to the general practitioners’ referral letter. Using the International Classification of Primary Care (ICPC) NPCD researchers recoded the registered diagnosis to an ICPC code [43]. If a patient entered through direct access (no referrer), the physical therapist registered the complaints and this physiotherapist’s diagnostic record was used and recoded by the researchers to an ICPC code. Patients were excluded if there was a possibility of recovery in the long term (e.g., fractures, ruptures, acute organ diseases, post-operative or pre-/post-partum diagnoses). To avoid the inclusion of non-chronic patients, the following diagnostic areas were excluded: symptom-related diagnoses (e.g., pain, stiffness, etc.), skin diseases, and physical deformities. Patients were excluded if no ICPC code was available. The sample selection is stated in Fig. 1.

### Variables

Therapists’ personality traits were measured using the Dutch version [40] of the BFI [39, 41] – a 41-item questionnaire using a 5-point Likert scale ranging from 1 (strongly disagree) to 5 (strongly agree) [40]. The BFI comprises five scales based on and named after the universally accepted personality trait dimensions Neuroticism, Extraversion, Agreeableness, Conscientiousness and Openness to experiences. These traits are known together as the Big Five [32, 44]. The term Big Five indicates that each domain represents a wide range of personality traits [39]. A higher score on Extraversion implied an ‘energetic approach towards the social and material world’; this includes being sociable, assertive, positive emotionality, active and talkative [39]. Higher scores on Agreeableness indicated a ‘pro-social and communal orientation towards others’ , including being sympathetic, forgiving, good-natured and polite. Conscientiousness indicated a ‘socially prescribed impulse control that facilitates task- and goal-directed behavior’. A higher score implied being reliable, well organized, self-disciplined and cautious. Neuroticism indicated ‘emotional stability and even-temperedness with negative–emotionality’. A lower score indicated being more calm, relaxed, secure and hardy. A higher score on Openness to experience indicated being more innovative, creative, curious and complex mentally and experientially [39]. The internal consistency of the BFI was high – Cronbach’s α ranged from 0.73 (Agreeableness) to 0.86 (Neuroticism) - and inter-scale correlation was relatively low (Fisher r-to-z transformation 0.24) [30, 45]. Convergent validity with the Big Five dimensions of Goldberg and the Neuroticism-Extroversion-Openness Five-Factor Inventory (NEO-FFI) was good [45]. The therapists’ life-changing event was seen as a possible confounder if the event appeared during the measuring period [46]. Therapists’ encounter with a life-changing event, either positive or negative, was answered with ‘yes’ or ‘no’ (e.g., getting married, bereavement, retirement, etc.) [46]. Other variables measured on therapist level were age, gender, education, and years of working experience.

The outcome of therapy was measured using the Numeric Rating Scale (NRS). The NRS is a widely used Dutch outpatient practice tool for evaluating treatment effect by looking at the course of complaints during treatment. Therapists recorded the NRS at the start and end of therapy. The NRS score ranged from 0.0–10.0, with a higher score indicating more severe complaints. Based on the NRS scores at the start and end of therapy, a difference score for the course of a patient’s complaint was calculated. A score of −10 to −1 indicated a decrease, a score of 0 indicated no difference and a score of 1 to 10 indicated an increase in the course of complaints. The test-retest reliability of the NRS is moderate in measuring pain [47] and high in measuring spasticity [48]. The validity is moderate to good in measuring a variety of patient-specific complaints [48–52]. A minimum clinically important difference was found to be 1.39 (SD 1.05) in measuring pain [47]. Other variables on patient level included patient’s age, gender, education, recurrence of complaint, duration of treatment and diagnosis.

### Sample size

The sample size was calculated per level, as different hierarchical levels (therapists and patients) were distinguished in the data [53]. The calculation was constructed using the following estimates: An Intraclass Correlation Coefficient (ICC) of 0.059 was estimated based on an average between-practitioners difference of 5.9 % [11, 54]. An average of six patients per therapist was estimated, based on LiPZ registrations of 2009. The variance was derived from a Z-score, as influences of personality traits on treatment outcome were unclear. A coefficient of 0.3 (conservative) was estimated, as previous research revealed diverse therapists’ effects (3–7 %) [11]. Based on these estimates, a power of 0.8 and significance level of 0.5, [54] the study needed to include 25 therapists and 152 patients.

### Data analysis

The computer software Stata 11 was used to analyse the data [55]. Categorical variables were presented as number and percentages. Continuous variables were presented as mean values with standard deviations or median values for non-normally distributed variables. Analyses of non-responders and missing data were performed using the Pearson’s Contingency coefficient Chi^2^, Independent *T*-test or Mann–Whitney *U* test. Unanswered BFI items (maximum of six per case per scale) were left out and the scale score was based on the remaining filled-in items [56, 57]. Differences between scale scores were checked using Cronbach’s α. Comparing Alphas between 1) scale scores including the remaining filled-in scores of the item with missing values and 2) the scale scores without the item that had missing values [58], the following was found: The Alpha of the scales stayed about the same – changing from 0.73 to 0.71 (Extraversion), 0.745 to 0.748 (Neuroticism), 0.76 to 0.77 (Conscientiousness), 0.6575 to 0.6581 (Agreeableness) and 0.723 to 0.718 (Openness to experiences). Based on the missing data analysis a full case analysis was performed [59–61].

Due to different hierarchical levels a two-level linear regression was performed. Multicollinearity was found to exist: therapist’s age was highly correlated to years of working experience (r = 0.94) [54, 58]. Therefore, only therapist’s age was included as more cases were available [62]. Not normally distributed variables were transformed into dummies. As the research question aimed at studying differences between therapists, a random intercept was used [63]. Regression was tested using the Wald test. Significant personality traits were entered with a fixed coefficient (Likelihood-ratio test = 0.58, *P* = 0.45) and regression coefficients were estimated using the Maximum Likelihood [63]. To avoid over-identification, the maximum number of variables included in the model was set to one variable per 10 therapists, and regression coefficients and significance levels were observed when entering a variable. The variables tested in the multilevel analysis are shown in Table 1.

**Table 1:** Variables used in analyses

Patient level			Therapist level		
Gender	Female	Categorical	Gender	Female	Categorical
(Female = 0)^a^	Male		(Female = 0)^a^	Male	
Age	Years	Continuous	Age	31–45 years	Categorical
			(31–45 years = 0)^a^	46–59 years	
				60–75 years	
Reoccurrence	No	Categorical	Life event	No	Categorical
(No = 0)^a^	Yes		(No = 0)^a^	Yes	
Education	Low	Categorical	Extraversion	Scale 1–5	Continuous
(Low = 0)^a^	Middle		Openness to experiences	Scale 1–5	
	High		Neuroticism	Scale 1–5	
	Other		Agreeableness	Scale 1–5	
			Conscientiousness	Scale 1–5	
Course of complaints	-10 − 10	Continuous			

^a^Reference value for dummies of ordinal or categorical variables

Low Primary School, Medium Secondary- or higher education, High University, Other not specified, yrs. Years

Variables were entered into the model using the forward method based on their univariate p-values (*p* = <0.10). First an empty model with the difference in course of complaint as dependent variable was calculated (Model 0). Next, patient variables were added in turn, to correct for effects on patient level (level 1) (Model I). Afterwards, personality traits (level 2) were added in turn (Model II). Next, therapist gender, age, life event, and remaining BFI variables, which were not significant in model II, were entered in turn. If the independent variable’s regression coefficient changed ≥10 % compared to model II the particular therapist variable was seen as a confounder and was included in the final model (Model III) [63]. Finally, the unexplained variance between therapists (Intraclass Correlation Coefficient, ICC) and the amount of variance that was explained by the therapist variables entered (R^2^) were calculated [63]. Subgroup analyses were performed to check the construct of the patient group used.

## Results

### Non-responding therapists and missing cases

Fifty-six therapists (77 %) completed the BFI questionnaire. The 17 non-responding therapists (23 %) did not significantly differ from the responding therapists with regard to gender (Chi^2^ = 0.30, *P* = 0.59), age (Z = 1.59, *P* = 0.11) but significantly for years working experience (Z = 2.03, *P* = 0.043). A total of thirteen BFI items (0.7 %) were not filled in; items were not mentioned twice. There were no significant differences between therapists who omitted an item and those who did not, regarding gender and age for Extraversion (respectively Z = −1.02, *P* = 0.31 and Z = −0.86, *P* = 0.39), Neuroticism (respectively Chi^2^ = 1.07, *P* = 0.30 and Z = 0.12, *P* = 0.90), Conscientiousness (respectively Chi^2^ = 0.01, *P* = 0.98 and Z = 0.87, *P* = 0.38), Agreeableness (Chi^2^ = 0.24, *P* = 0.63) and Openness to experiences (respectively Chi^2^ = 0.49, *P* = 0.48 and Z = −0.53, *P* = 0.59).

In the patient cases without an ICPC code there was no difference between missing and completed patient cases with regard to patient’s gender (Chi^2^ = 1.93, *P* = 0.17), age (Z = 0.34, *P* = 0.73) and significant difference in education (Z = −3.17, *P* = 0.002).

### Characteristics

Thirty-nine therapists and 393 patients were included in the analysis. Therapists had an average age of 53 years (SD 1.6, range 28–69) and were mainly male. They had worked on average 27 years (SD 1.4, range 4–40). Besides being a general physiotherapist, therapists were specialized in the pelvis (*n* = 2, 5 %), paediatrics (*n* = 2, 5 %), manual therapy (*n* = 10, 26 %) oedema (*n* = 1, 3 %), sport (*n* = 4, 10 %) and/or other specializations (*n* = 4, 10 %). The therapists treated an average of 10 patients with CD within the three-year period (range 1–51). The BFI scores were generally higher on Openness to experiences (mean 3.42, SD 0.09), Extraversion (mean 3.49, SD 0.07), Conscientiousness (mean 3.69, SD 0.08) and Agreeableness (mean 3.75, SD 0.06) and lower on Neuroticism (2.39, SD 0.09). The range of all but one trait (Neuroticism) was limited. Therapists’ characteristics are shown in Table 2.

**Table 2:** Descriptive statistics of the physiotherapists (*n* = 39)

Therapist variables		Outcome
Gender, *n* (%)	Female/Male	10 (26)/29 (74)
Age (yrs.), *n* (%)	≤30	1 (2.5)
	31–45	6 (17)
	46–59	23 (57.5)
	60+	9 (22.5)
Education^a^, *n* (%)	Specialization	9 (23)
	Academic Education (MSc.)	2 (5)
	Course aimed at chronic patients	12 (30)
	Course aimed at communication & coaching	15 (38)
	Course aimed at self-management	7 (18)
	None of above	13 (33)
Life-changing event ≤3 years., *n* (%)	Yes/No	19 (52)/17 (47)
Big Five, mean (min – max)	Neuroticism	2.38 (1.25–3.88)
	Extraversion	3.49 (2.63–4.63)
	Agreeableness	3.75 (3.00–4.78)
	Conscientiousness	3.69 (2.89–4.89)
	Openness to experiences	3.42 (2.70–4.80)

% Percentage, *n* number, min minimum, max maximum, SD standard deviation, Yrs. Years

^a^more than one answer possible

Patients’ average age was 67 years (SD 15, range 18–98) and they were mostly female. Overall, the patients experienced a clinically important reduction in their complaint (Mean −3.66, SD 2.5, −9 min – -2 max). The most frequent diagnosis was Osteoarthritis disorders (*n* = 180, 46 %), followed by Rheumatoid Arthritis (*n* = 40, 10 %) and Cerebral Vascular Accident (*n* = 39, 10 %). Patients’ characteristics are shown in Table 3.

**Table 3:** Descriptive statistics of patients (*n* = 393)

Patient variable			Outcome
Gender, *n* (%)		Female/Male	240 (61)/153 (39)
Age yrs., *n* (%)		≤30	9 (2.3)
		31–45	22 (5.6)
		46–59	81 (20.6)
		60–75	154 (39.2)
		76–85	99 (25.2)
		≥86	28 (3.1)
Education, *n* (%)		Lower	143 (36.3)
		Middle	83 (21.1)
		Higher	46 (11.7)
		Other^a^	121 (31)
Recurrence of the complaint, *n* (%)		Yes	139 (36)
		No	250 (64)
Severity, mean (SD, 95 % C.I.)		Start therapy	6.84 (0.1, 6.6–7.0)
		End therapy	3.19 (0.1, 2.9–3.4)
Disease, *n*	Cancer	Neoplasm or lymphatic system	1
		Esophageal malignancy	1
		Nervous system	1
		Neoplasm bronchus/lung	1
	Cardiovascular	Heart failure	2
		Heart valve disease	2
		Cerebral ischemia	1
		Cerebrovascular accident	39
		Claudicatio intermittent	18
	Rheumatic disorders	Fibromyalgia	15
		Rheumatoid arthritis^b^	40
		Other arthritis	26
		Tietze syndrome	4
	Degenerative bone and joint disorders	Osteoarthritis of the Spine	76
		Osteoarthritis of the Hip	34
		Osteoarthritis of the Knee	70
		Osteoporosis	16
	Disorder (central) nervous system	Multiple sclerosis	6
		Parkinson	15
		Alzheimer disease	2
	Lung diseases	Chronic bronchitis	2
		Emphysema/COPD	17
		Asthma	2
	Metabolic disorders	Cystic fibrosis	1
		Diabetes Mellitus	1

^a^Filled in by therapist as other, % percentage, n number, SD standard deviation, yrs. Years

^b^(incl. rheumatic polymyalgia), CI Convenience Interval

% percentage, n number, SD standard deviation, X mean, yrs. Years

### Multilevel analysis

The analysis is shown in Table 4.

**Table 4:** Steps to prediction model for the course of complaints

		Coef.	S.E.	Z	*P*	95 % CI	ICC
Model 0							
Intercept		−3.66	0.19			−4.02 – -3.30	
Total Model							
Var. Th. level		0.47	0.31			0.13–1.73	0.076
Var. Pt. level		5.75	0.43			4.96–6.66	
Model I							
Patients							
	Gender	−0.47	0.25	−1.88	0.060	−0.96–0.02	
	Age	0.01	0.01	1.76	0.079	−0.002–0.03	
Intercept		−2.90	0.44			−3.76 – -2.03	
Total Model							
Var. Th. level		0.41	0.29			0.11–1.63	0.067
Var. Pt. level		5.68	0.43			4.90–6.58	
Model II							
Patients							
	Gender	−0.48	0.25	−1.94	0.053	−0.97–0.006	
	Age	0.01	0.01	1.63	0.103	−0.003–0.03	
Therapists							
	Neuroticism	0.59	0.32	1.81	0.070	−0.048–1.22	
Intercept		−4.27	0.88			−5.99 – -2.56	
Total Model							
Var. Th. level		0.36	0.26			0.09–1.46	0.060
Var. Pt. level		5.65	0.42			4.88–6.54	
Model III							
Patients							
	Gender	−0.43	0.25	−1.66	0.098	−0.92–0.08	
	Age	0.01	0.01	1.11	0.269	−0.01–0.03	
Therapists							
	Neuroticism	0.71	0.29	2.47	0.014*	0.15–1.28	
	Gender	0.72	0.32	2.21	0.027*	0.08–1.35	
	Life events	−0.54	0.32	−1.68	0.092	−1.16–0.09	
Intercept		−5.42	0.94			−7.27 − -3.57	
Total Model							
Var. Th. level		0.12	0.19			0.01–2.57	0.021
Var. Pt. level		5.60	0.43			4.82–6.52	

*Significant variables ≤0.05, CI convenience interval, coef. Regression coefficient, ICC Intraclass Correlation Coefficient, *P* significant level, Pt Patient, R^2^ percentage of variance explained by model, ^S^E. standard error, Th Therapist, Z z-score

Of the initial model 7.6 % (ICC 0.076) was ascribed to inter-therapists variation (Model 0, Table 4). The patients’ gender (*P* = 0.06) and age (*P* = 0.08) were found to be eligible and were entered into the model (Model I, Wald Chi^2^ = 6.71, *P* = 0.03). The ICC was reduced to 6.7 %, meaning that a small part of the variance (9 %) between therapists was explained by these patient variables.

Of the Big Five variables, only Neuroticism was found to be eligible (Model II, Wald Chi^2^ = 10.11, *P* = 0.02). Therapist gender and experienced life events were added as confounders (Model III). Neuroticism was found to be significant (Wald Chi^2^ = 16.82, *P* = 0.005). Table 5 describes how the R^2^ was calculated. 70 % of the variation between therapists could be explained by Neuroticism, therapist gender and experienced life events.

**Table 5:** Amount of explained variance per model

Model			R^2^
_Total_ R^2^	(0→I)	$\frac{\left(0.47-5.75\right)-\left(0.41+5.68\right)}{\left(0.47+5.75\right)}$	=0.13
_Therapist variables_R^2^	(I→III)	$\frac{0.41-0.12}{0.41}$	=0.71
_Patient variables_R^2^	(0→I)	$\frac{5.75-5.68}{5.75}$	=0.01

The subgroup analysis using only patients with Osteoarthritis (*n* = 180) treated by 30 therapists showed similar results to the main model, with Neuroticism as the independent variable and Conscientiousness and therapists’ gender as confounders: constant F = −10.18, Neuroticism F = 1.15, *p* = 0.003 (0,40–1.91 95 % CI), Conscientiousness F = 0.68, *p* = 0,07 (−0,04–1.41 95 % CI), therapists’ gender F = 0.76, *p* = 0.55 (−0.02–1.54 95 % CI). This might give an indication that the kind of chronic disease is unrelated to the influence of therapist on treatment outcome.

## Discussion

The purpose of this study was to explore the influence of therapists’ personality traits on treatment outcome in patients with CD. Specht et al. [46] indicated that personality can change not only change due to maturation, [64] but also due to social demands and experiences. These changes are more pronounced at younger and older ages, but occur throughout a person’s lifetime [46]. As personality traits might be accounted for, knowledge of traits that influence treatment outcome might be useful for general training of therapists and specifically for patients with CD. Generally, the results indicate that Neuroticism might have an influence on treatment outcome in patients with CD. A higher score on Neuroticism was associated with worse treatment outcomes. The current variables Neuroticism, gender and life events, explained approximately 71 % of the total variance between therapists. Therefore future research looking at the differences between therapists in treatment outcome should include the identified variables. Of the Big Five trait, Neuroticism was the only personality trait that was associated with better treatment outcomes. This suggests that treatment by therapists who tend to be calmer, more relaxed, secure and hardy, may produce better treatment outcomes in patients with CD.

To the author’s knowledge, this is the first study that looks systematically at physiotherapists’ personality traits in relation to treatment outcome. The indication of the possible relevance of Neuroticism corresponds with evidence found in the field of psychotherapy, showing that being treated by secure therapists predicts a better outcome [25]. Moreover, the overall ICC of 0.075 found in this study is similar to previous research showing an ICC of 0.03–0.07 on therapist level [11]. The results are based on a sample of predominantly older women with chronic diseases, treated by older male therapists. Therefore caution should be exercised when generalizing the current results. More research into the influence of these traits on treatment outcome in a more heterogeneous sample is needed. Evidently, this study supports prior research that a physiotherapist effect does exist [11].

Contrary to expectations, no evidence was found for the four other personality traits. This finding contradicts previous research in psychotherapy suggesting that traits including being empathic, [25–27, 29] cautious, non-intrusive, [25] respectful, being able to adjust and exuding warmth [29] (as a psychotherapist or general practitioner) improve treatment outcome. The contradiction with earlier research might be due to limited distribution of the personality traits and the difference in professions and diagnosis being examined. Further research with a sample of therapists with a wider range of Big Five scores is needed to obtain a better understanding of the influence of all Big Five traits. The influence of therapists’ gender confirmed the results of another physiotherapy study that investigated the placebo effect and its relation to personality [28]. The study indicated that a female therapist was associated with better outcomes in patients with an irritable bowel syndrome.

While little is known about the influence of being more neurotic as a therapist on patient outcome in research, more is known of the influence on the therapist himself. Studies in the fields of psychotherapy and general practitioners underline that being less neurotic reduces the practitioner’s chances of emotional exhaustion (a form of burn-out) [65] and increases their sense of satisfaction with life [66]. If a therapist does not feel mentally stable, it is reasonable to assume that this might have consequences for his or her attitude when interacting with the patient. Further research is needed to clarify these assumptions.

Reflecting on ones personality as a physiotherapist could yield information on the existence of negative influencers, like Neuroticism. In the fields of psychotherapy and general practice, training has been advised as part of the professional education [67]. Tools like communication skills training might be used as supplement to reflection, [68] but the authors believe that self-awareness and reflection training during the early stages of study are needed, before these tools can be used effectively.

Other mechanisms such as patient personality traits, health beliefs, moral compass, placebo effects and other interaction mechanisms might affect both the patient and the therapist and therefore treatment outcome [69]. For example, the patients’ beliefs regarding the effect of treatment or previous experiences with their goal of ‘getting physically active’ might influence their motivation towards adopting a more active role in the self-management process, which could influence treatment outcome [69]. In the same way, a therapist who experienced negative results when engaged in physical exercise may have created a different conceptualization of the goal ‘getting physically active’. This, combined with having a certain personality trait, like being more neurotic, might increase the chance of a negative outcome when getting others to be physically active. Future studies that focus on the physiotherapist’s effect on treatment outcome ought therefore to not only look at the personality domains as such, but also take other mechanisms like experiences, health beliefs, etc. into consideration.

There are implications that CDs influence patients’ wellbeing differently [70, 71]. For example, it is known that anxiety and depression are common in patients with Chronic Obstructive Pulmonary Diseases [72]. Consequently, knowledge of personality traits that influence treatment outcome in specific CD groups would support therapists during treatment as they could adjust their approach accordingly. Therefore, analysis of specific CD groups might be of interest. In the current study, the outcome in the subgroup analysis points to patients with Osteoarthritis, showing that both Neuroticism and Conscientiousness are possible influencing factors. The association between Conscientiousness and Neuroticism has been described in previous studies [22, 23].

When investigating the therapist’s effect, interdependency of the cases have to be taken into account as this can change the outcome considerably [63]. A multilevel analysis, especially including subgroup analysis, requires large sample sizes. This can be a hindrance when performing this type of analysis. The current study gives an example of the use of longitudinal electronic patient record data for multilevel research into the physiotherapist effect. The use of the NPCD database reduced the organizational burden considerably, particularly in view of the number of therapists and patients needed. Furthermore, the database provided standard patient care data. Accordingly, missing patients were not study-specific and therapists were not aware of the patient data researched for this study.

### Limitations

Unfortunately, in the NPCD database, around 60 % of the outcome variable was missing, causing a loss in the number of patients and therapists that could be studied. The missing data in the patient database was due to the fact that the study was based on voluntary registration of some of the variables in the NPCD. The authors did compare the missing data with the existing data. The demographic data did not differ significantly between missing and non-missing patients and therapists’ cases. Despite the amount of missing data, there were enough patients and therapists included to perform the analysis and there was a higher average of patients treated per therapist than estimated (ten vs. six) for the patient sample size. For the therapist data, the authors did try to reduce non-responsiveness by sending two reminders. It could be that a specific group of therapists, with specific personality traits, did not respond. However, there was variation in the BFI scales, albeit low. Therefore no large effect of missing a subgroup is expected.

Although the authors tried to account for the influence of a life event on personality traits [46], it was not specified if the experience was positive or negative. As the effect can be the opposite depending on the experience, no judgement can be made on the kind of influence the item life events has on Neuroticism [46]. Further research is needed to study this in greater depth.

Personality inventories like the NEO-FFI might possibly have been more precise for measure personality traits [45]. That said, the BFI was chosen for practical reasons, since it does not take too long for therapist to fill out. Besides, the BFI provides a general view on personality, which was the purpose of the study.

## Conclusion

There are indications that patients with CD who are treated by therapists who tend to be calmer, more relaxed, secure and hardy have a greater reduction in severity of complaints compared to patients treated by therapists who show less of these traits. Being a male therapist and having experienced life events influence the outcome positively. However, more extensive research is needed to validate the current findings.
